# Digit Tip Injuries: Current Treatment and Future Regenerative Paradigms

**DOI:** 10.1155/2019/9619080

**Published:** 2019-01-21

**Authors:** Travis J. Miller, Peter L. Deptula, Gregory M. Buncke, Zeshaan N. Maan

**Affiliations:** ^1^Department of Surgery, Division of Plastic and Reconstructive Surgery, Stanford University School of Medicine, Stanford, CA 94304, USA; ^2^The Buncke Clinic, San Francisco, CA 94114, USA

## Abstract

Over the past several decades there has been a profound increase in the understanding of tissue regeneration, driven largely by the observance of the tremendous regenerative capacity in lower order life forms, such as hydra and urodeles. However, it is known that humans and other mammals retain the ability to regenerate the distal phalanges of the digits after amputation. Despite the increased knowledge base on model organisms regarding regenerative paradigms, there is a lack of application of regenerative medicine techniques in clinical practice in regard to digit tip injury. Here, we review the current understanding of digit tip regeneration and discuss gaps that remain in translating regenerative medicine into clinical treatment of digit amputation.

## 1. Introduction

The human hand plays numerous critical roles in everyday function. The hand is used for labor, sensation, communication, and intimacy; injury to the hand profoundly affects almost every aspect of a person's life. The anatomy of the hand is complex and consists of multiple tissue types, including bone, tendon, nerves, blood vessels, and skin. Injury to any of these tissues can cause significant functional impairment. The care of hand injuries thus requires specialized treatment by practitioners who have undergone advanced training in the field of hand surgery [1]. Despite the treatment of hand injuries in high volume centers by experienced multidisciplinary teams, it is incredibly rare for the natural anatomy of the hand to be restored after a debilitating injury. Even with rigorous occupational therapy, permanent dysfunction can result [2]. Since the function of the hand relies on its intricate anatomy, complete restoration of injured tissues would be the ideal treatment to preserve the functional capacity of the hand.

In contrast to urodeles, such as salamanders and newts, which maintain life-long capacity for epimorphic regeneration of injured tissue by the formation of a stem cell blastema, mammals are limited in their ability to regenerate tissue after the prenatal period [3]. Other than in select tissues containing functionally relevant stem cells, such as the liver, bone marrow, and intestinal mucosa, epimorphic regeneration is replaced by a fibrotic “patch” response in adult mammals [4–6]. This patch repair restores the barrier between the body and the external environment but is largely devoid of native tissue properties. This process can lead to unsatisfactory functional outcomes [3, 7].

The switch from regenerative to fibrotic healing responses in the developing human mirrors how regenerative capacity diminishes with the evolution of higher organisms. Planarians can regenerate almost their entire structure while the regenerative capacity of the hydra is so great that they are believed to be biologically immortal [8]. Salamanders and newts, which are structurally more complex, can still regenerate entire limbs and tails, restoring preinjury structure and function [9, 10]. Epimorphic regeneration of an extremity in mammals, however, is significantly more limited, and the fibrotic reaction predominates. There is great interest in understanding the cellular and molecular mechanisms of regeneration seen in lower eukaryotes in hopes of “reawakening” them in human extremity injuries. Unfortunately, current clinical treatments for extremity injuries are unable to harness the lost capacity for epimorphic regeneration. Here, we review the current understanding of extremity regeneration and explore clinical approaches to the care of hand injuries in the context of regenerative treatment paradigms.

## 2. Paradigms of Digit Tip Regeneration

### 2.1. Cellular Signaling and Necessary Tissue Units

Tissue regeneration has been observed in mammals, including the ears of rabbits and the antlers of deer, demonstrating that higher eukaryotes are also capable of tissue regeneration [11, 12]. In these higher eukaryotes, a regenerative blastema, consisting of a mass of heterogeneous, lineage-restricted stem and progenitor cells, forms at the site of injury [13, 14]. These proliferating cells then differentiate to replace missing tissue. The mouse limb provides a valuable model for understanding the formation of blastema and subsequent digit tip regeneration in mammals, which offers promise in developing treatments in humans [15, 16]. In fact, humans appear to display similar capacity to mice for spontaneous digit tip regeneration [17, 18].

**Figure 1:**
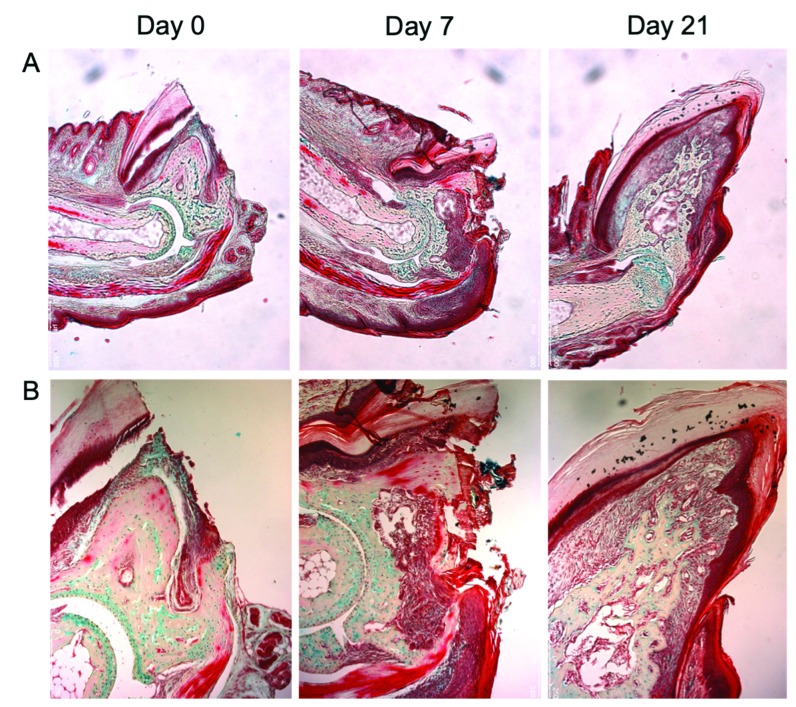
Regenerating adult mouse digit after sharp amputation through P3. By day 21, the digit structure and all of its native tissue types are restored. (a) 5x magnification; (b) 10x magnification.

In the mammalian limb, only the digit tip in mice and primates (including humans) is capable of spontaneous regeneration into adult life [19–21]. In all of these organisms, regeneration has been observed in amputations involving the distal phalanx (P3) only and not more proximally. Successful regeneration of the digit tip is also level specific; total regeneration does not occur in the mouse model if less than 60% of the proximal P3 remains after amputation [16].  Figure 1 demonstrates the regenerative capacity of the adult mouse digit tip. The tissues in this area consist of skin, blood vessels, fat, bone, and tendon which are seen throughout the digit. However, a specialized nail bed unit is also present and is required for the regenerative response [22]. It is crucial to note that the nail is a specialized organ that replenishes itself throughout human life. Wnt signaling is necessary for nail replenishment, and Wnt's role in embryonic development is well characterized [22, 23]. The germinal matrix of the nail bed contains Wnt-active nail stem cells (NSCs). The reaction of these NSCs to amputation is required for the formation of the regenerative blastema with Wnt signaling in the ectoderm appearing critical for a mesodermal response [22]. Indeed, transplanting the nail bed to the site of a more proximal amputation, which would otherwise undergo fibrotic healing, results in ectopic bone growth as part of a regenerative response [24]. Even in the absence of traumatic injury, epithelium-derived Wnt is necessary for the maintenance of the underlying digit bone, highlighting the importance of this signaling pathway in governing ectodermal-mesodermal interactions in the digit tip [25]. Leucine-rich repeat-containing G protein-coupled receptor 6 (LGR6), a known agonist of the Wnt pathway, has also been identified as a marker for nail stem cells, and its loss prevents a regenerative response [26]. The LGR family of proteins, which acts as receptors for R-spondins, activate Wnt signaling during embryogenesis. Their role in maintaining adult stem cells is currently under investigation [27].

**Figure 2:**
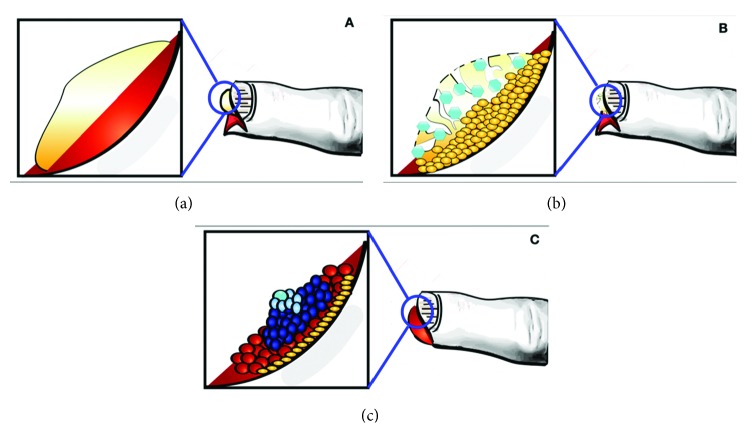
(a) Exposed bone and soft tissues following fingertip amputation through the distal phalanx. (b) Exposed bone undergoes histolysis while epidermal cells begin to cover exposed tissues. (c) Blastema of rapidly proliferating cells develops at the site of tip injury, beginning the process of digit tip regeneration.

In the regenerating digit tip, the blastema, derived from the resident stem and progenitor cells, forms at the wound site with proliferation of cells at an accelerated rate [28–30]. The identification of the blastema-specific cells following amputation is complicated by a concurrent histolytic response. It has been demonstrated that a period of tissue breakdown and closure of the wound with epidermal-derived cells precedes regeneration [13, 14, 22]. This process is schematized in Figure 2.

During the transition between histolysis and blastema formation, critical cell signaling processes occur. Transforming growth factor beta (TGF-*β*), which has a known role in fibrotic wound healing, is required to initiate and modulate the regenerative response. Experimental inhibition immediately after amputation prevents downstream activation of bone morphogenic protein (BMP) and extracellular signal-related kinase (ERK) signaling pathways [31]. TGF-*β* regulates wound epithelium formation after amputation, the establishment of regenerating tissue units, and cell proliferation in the blastema [32]. TGF-*β* also appears to play a role in the regulation and activation of local stem cells in response to environmental perturbations, and its activation is spatial and temporally sensitive [33]. Matrix metalloproteinases (MMPs), in addition to promoting extracellular matrix degradation and remodeling, also have been found to promote TGF-*β* activation [32, 34]. Likely, MMPs become activated by both mechanical and chemical stimuli at the time of amputation, enabling TGF-*β* activation and subsequent regulation and transition between histolysis and blastema proliferation [35]. Since TGF-*β* is also a strong agonist of the fibrotic healing pathway, tight regulation and timed inhibition in regeneration processes is necessary. The complex regulation of TGF-*β* is a substantial focus of ongoing research [36].

Once the blastema has formed, the cells within it express MSX1, a transcriptional repressor that is necessary for regeneration and acts through upregulation of BMP4 [14, 37]. In more proximal amputations, the addition of BMP has been found to partially rescue the regenerative response [37, 38]. Signaling pathways that repress cell differentiation and promote proliferation are also necessary for successful regeneration. Additional blastema cell markers include pigment epithelium-derived factor (PEDF) and chemokine receptor type 4 (CXCR4), which is the receptor for stromal cell-derived factor 1 (SDF1) [39]. LGR6 has also been identified in the blastema, and as noted previously, its deletion precludes a regenerative response [26]. Sonic hedgehog (Shh), whose role is well-described in anterior-posterior patterning in growing limb buds, is also expressed in the blastema and plays a similar role in epimorphic regeneration patterning, though its regulation is not fully understood [40]. Its role complementing FGF has also been explored, and experiments comparing anterior and posterior blastema with differential expression of FGF confirm that coexpression of SHH and FGF are necessary to drive regeneration to completion [41].

While there are many similarities between embryonic development and adult digit tip regeneration, there are also some distinct differences. In adult regeneration, the formation of the P3 bone by blastema cells occurs via direct ossification without a chondrogenic intermediary, which differs from the endochondral development of the P3 in the embryo. Thus, the new bone that is formed is trabecular in nature and is not an exact replica of the amputated part that consisted of cortical bone. This bone has a higher volume than preinjury bone but over time regains the tapered morphology of the native P3 [29]. The palmar-dorsal anatomy undergoes restoration of the volar fat pad, nail curvature, and associated paronychial folds. This patterning is likely governed by engrailed-1 which is expressed in the regenerating tip, but this pathway requires further investigation [13].

As noted previously, NSCs and Wnt signaling in the ectoderm have been found to be necessary for digit tip regeneration. This is in part due to the effects of Wnt on nerve growth and reinnervation of the regenerating digit tip. Lack of innervation in the blastema influences FGF2 expression in nail epithelium and results in patterning defects in the bone and nail matrix, consistent with the embryologic role of the FGF family during limb development [22, 42–44]. The interstitial, fibroblastic, and perineurial cells of the remaining tip also likely play a crucial role in the regeneration of blood vessels, recruitment of smooth muscle cells, neural regeneration, and reformation of connective tissue. However, the activation of these cells in the context of regeneration is poorly understood [39, 45].

### 2.2. Variance in Regeneration across Organisms

It seems that though certain anatomical units are needed for regeneration to occur, these units are not necessarily conserved across species. For example, salamanders are the poster child of limb regeneration but do not have fingernails, which are required for regeneration in mammals. Some similarities in limb regeneration do exist, as peripheral neural innervation has been shown to be a requirement for regeneration in fish, salamanders, and mice [42, 46, 47]. In mammals, this is partially due to nerve-associated Schwann cell precursors that stimulate blastema growth by secreting oncostatin M and platelet-derived growth factor AA, and though the protein secreted differs in salamanders, Schwann cells again play a critical role [48, 49]. Interestingly, directing a transected nerve to a site of injury in an axolotl can produce supernumerary limbs [50]. Despite the variations seen, the role of the peripheral nervous system is preserved across multiple species, highlighting its importance when considering regenerative strategies.

Classically, it was felt that the blastema present in all animal models of regeneration consisted of multipotent stem cells and that these stem cells likely formed the blastema after dedifferentiation from mature tissues at the injury site [51–54]. More recent research in multiple animal models suggests that cells participating in digit tip regeneration are fate-restricted and do not have a uniform origin across different animals. In zebrafish, osteoblasts “dedifferentiate” and enter a proliferative state to replace amputated bone, but these cells do not express markers of multipotency [55]. Resident stem cells do play a role in mice, however, as the regenerating osteoblasts are derived from stromal and bone marrow cells, but again they are fate-restricted [56, 57]. It is interesting to note that even in the same animal order, the cellular processes of regeneration differ; in newts, the blastema consists of dedifferentiated cells derived from various tissues, while in axolotls, blastema formation involves satellite stem cell activation [58]. Notably, at a fundamental cellular level, the requirements and characteristics of regeneration are still being elucidated and may be unique to particular organisms. Many of the paradigms seen in animal models may differ from that in humans. These differences, however, also suggest that multiple strategies may be effective for regenerating human extremities and further understanding of mammalian digit tip regeneration would be useful for translational application.

## 3. Current Clinical Approaches to Digit Tip and Upper Extremity Injuries

### 3.1. Wound Closure and Regeneration

There are numerous tactics for the treatment of the amputated digit tips. In terms of achieving spontaneous regeneration, the most effective strategy of treatment is, perhaps ironically, pursuing conservative management. The most successful cases are seen in children, where amputations left open have been found to spontaneously lengthen and regain the nail plate and contour [17]. However, there have been reports of total regeneration in adults as well [19]. In contrast to mice where the critical level of amputation permitting regeneration is within the nail plate, complete regeneration in humans has been described proximal to the nail fold, though distal to the distal interphalangeal joint [16, 18, 59]. One trial by Das and Brown compared nonintervention to other closure methods in children under 12. The study noted patients who only underwent dressing changes had the best aesthetics, nail contour, and 2-point discrimination [60]. Champagne et al. advocate wound care for all amputations distal to the germinal matrix and have even proposed a classification system based on the level of amputation to aid clinicians in this decision [61]. There are downsides to this conservative approach, however. Open wounds are a source of ongoing pain for the patient, and regular dressing changes are needed at the amputation site for up to 12 weeks until wound closure is achieved. This may not be tolerable to many patients or feasible depending on their social situation or ability for self-care.

Often, clinicians will choose to perform closure of a distal amputation site. This presents many advantages for the patient as wound closure is achieved immediately. For example, in manual laborers, immediate closure may allow earlier return to work. Closure can be performed directly with or without revision amputation/bone shortening by using local skin flaps or tissue grafting (sometimes with the amputated part itself). A systematic review by Yuan et al. showed that 91% of patients undergoing revision amputation with or without local flaps were satisfied with their result and maintained an acceptable range of motion [62]. However, the closure of skin flaps has been shown to inhibit the regenerative response. This has been demonstrated in salamanders and newts, where whole limbs can often be regenerated when stumps of these organisms are left open, closure of the skin blunts regeneration [63]. Further investigation suggests that alteration of ionic currents in the limb occurs with skin closure, and this impedes regeneration [64]. The placement of a skin flap also places basement membrane over the wound, where in the regenerating limbs only an epidermal layer forms and then the underlying stem cell blastema develops. Animal models show that the basement membrane placed over wounds may inhibit the molecular interactions needed for regeneration [65]. At the time of this review, there has been little to no work in humans regarding the electrochemical dynamics and basement membrane effect in human amputees. However, from the authors' experience, spontaneous lengthening does not occur after the closure of a digit tip wound, and it is likely that these mechanisms play a role in inhibiting digit tip regeneration in humans.

**Figure 3:**
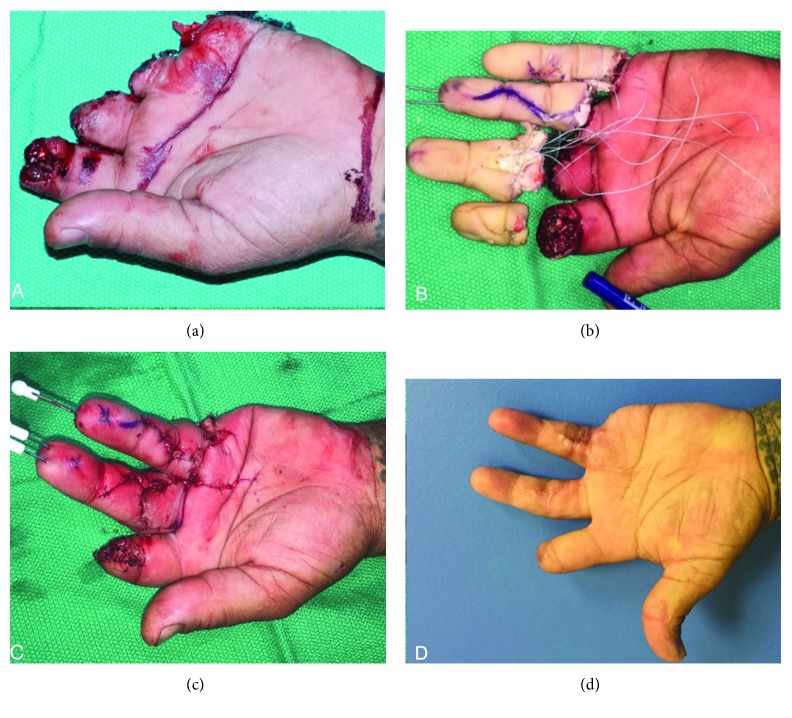
Multiple digit replantation. Panel (a) shows the initial injury, and panel (b) shows the digit parts for attempted replantation with tendons tagged. The index finger was amputated too distal to allow replantation, and the small finger part was too mangled to allow microsurgical anastomosis; revision amputation was employed in these fingers. The immediate postoperative photo is seen in panel (c). Final healed result is seen in panel (d).

In many cases, replantation of an amputated digit tip with vessel microanastomosis is possible and can have excellent cosmetic and functional results. For many injuries, this is considered the gold standard of treatment; a retrospective review by Hattori et al. demonstrated that replantation patients have a higher functional level and satisfaction compared to revision amputation [66]. However, even though native tissue is replaced with replantation, outcomes rarely match preinjury function. The healing in replants follows traditional paradigms, which means scar formation where soft tissue is reunited. Scar adhesions, especially involving the flexor and extensor tendon mechanisms, lead to postoperative stiffness. This is complicated by the fact that bone must heal (a period of 4-8 weeks) to allow a stable base for the digit before a range of motion therapy can begin to counteract adhesion formation [67]. Even with maximal therapy and scar lysis procedures, patients are often unable to achieve an arc of motion comparable to the native finger [2, 68]. Patients also rarely have a total recovery of nerve function, and many do not regain useful 2-point discrimination [68]. Even with the most optimal outcomes, the patient must undergo a period of recovery lasting up to 1-2 years, which leads to a significant time off of work and thus a large societal cost [69]. The act of finger replantation is also technically demanding and can only be reliably performed at replant centers with specialized surgical teams. Given the time sensitivity of replantation (with cold ischemia time <12 hours preferable to ensure amputated part viability), replantation is also not always feasible for patients in rural areas [70]. The mechanism of injury may also preclude surgical reattachment. Replantation thus has many limitations in restoring both anatomy and function in amputees.  Figure 3 demonstrates a reasonable result for a patient who sustained avulsion-type amputation of multiple digits with replantation as part of his treatment; it is notable that even with the most up to date treatments, preinjury form and function are not completely restored.

### 3.2. Possible Adjuncts in Digit Tip Regeneration

The application of 2-octyl cyanoacrylate is a common clinical practice. In the mouse model, the use of tissue adhesive accelerates wound closure and attenuates the histolytic response, which may preserve native tissue and decrease the burden of tissue required to regenerate [71]. However, in humans, the use of a topical adhesive to achieve closure for an amputation is not feasible given digit size. Additionally, the use of the adhesive is only approved for epidermal approximation, not on exposed deeper structures [72]. However, the use of tissue adhesive has been shown to be useful for nail bed repair [73, 74]. A series describing the use of tissue adhesive to achieve hemostasis in digit avulsion has been described, but long-term outcomes for these patients were not followed [72].

The utility of hyperbaric oxygen in digit tip amputation has also been explored. In the mouse model, the amputation stump changes from a high oxygen tension environment to a hypoxic environment within the blastema [75]. Notably, VEGFA is not expressed in the regenerating digit tip, and VEGF treatment actually inhibits regeneration [76, 77]. In mice, treatment with daily hyperbaric oxygen after digit amputation seems to prolong the histolysis phase and the proximal extent of bone degradation; therapy also maintains regeneration competency with more proximal P3 loss [78]. The role of hyperbaric oxygen therapy has been explored for the treatment of poor wound healing and infection in humans, but its use in digit tip amputation treatment has not been documented [79]. Given the effects seen in mice, a trial of hyperbaric oxygen therapy in human digit tip injuries is needed. Current clinical recommendations call for the closure of wounds that exceed 1.0-1.5 cm^2^, which will arrest regenerative potential; hyperbaric oxygen could potentially accelerate and/or strengthen the regenerative response in wounds that otherwise would not be acceptable to leave for healing by secondary intention [80].

Treatment of amputated digit tips with recombinant signaling proteins has also not been explored. Conceptually, it is feasible that with the appropriate application of signaling molecules, the regenerative pathway could be activated and/or optimized following digit injury. Recombinant bone morphogenic protein is now used in clinical practice for orthopedic and dental procedures when bone gaps are encountered. Its role in digit tip regeneration is known, but it has not yet been applied for digit tip injuries [81]. The R-spondin family of proteins, which are expressed in embryogenesis, are also potent activators of adult stem cells *in vivo* and *in vitro*; R-spondins thus have a potential therapeutic role in regenerative medicine that requires further study [82]. Though basic research continues to elucidate the complexities of signaling proteins in the human regenerative pathway, clinical protocols investigating these molecules are also required.

Tissue engineering is also a possible intervention to harness regenerative pathways for digit tip injuries. The use of a tissue scaffold and repopulation with stem cells has shown promise in engineering liver tissue [83]. However, the repopulation of a digit tip scaffold presents numerous problems. One is the diversity of tissue types to reconstitute; skin, bone, fat, muscle, tendon, and nerve are all integral to digit tip function, and the complexity of repopulating all of these tissue types is daunting in contrast to other organs. It is also unknown what type of scaffold would be necessary to promote regeneration. Decellularized nerve allograft has shown favorable outcomes and supports the idea that a structural matrix can support regrowth of native nerve tissue, but adequate scaffolds for the other tissue types in digit tips are lacking [84]. A better understanding of the critical lineage-restricted progenitor cell populations is involved, and the ideal matrix to support patterning and differentiation is necessary to guide tissue engineering approaches to digit tip regeneration.

## 4. Conclusions

While much is understood regarding the paradigms of regeneration, the use of regenerative medicine remains lacking in hand and digit injuries, despite the conservation of digit tip regeneration in humans. The treatment that is most conducive to tip regeneration is actually no treatment at all; healing by secondary intention has the capacity to restore the original length and function of the digit, though this treatment is limited only to the distal phalanx level. Further study is needed to develop clinical techniques that effectively harness the regenerative capacity of the human digit tip for more proximal limb injuries.
